# Complex N-glycosylation of mGluR6 is required for trans-synaptic interaction with ELFN adhesion proteins

**DOI:** 10.1016/j.jbc.2024.107119

**Published:** 2024-02-28

**Authors:** Michael L. Miller, Mustansir Pindwarawala, Melina A. Agosto

**Affiliations:** 1 Faculty of Science, Medical Sciences Program, Dalhousie University, Halifax, Nova Scotia, Canada; 2 Retina and Optic Nerve Research Laboratory, Dalhousie University, Halifax, Nova Scotia, Canada; 3 Department of Physiology and Biophysics, Dalhousie University, Halifax, Nova Scotia, Canada; 4 Department of Ophthalmology and Visual Sciences, Dalhousie University, Halifax, Nova Scotia, Canada

**Keywords:** metabotropic glutamate receptor, mGluR6, synapse, N-linked glycosylation, retina, bipolar cell

## Abstract

Synaptic transmission from retinal photoreceptors to downstream ON-type bipolar cells (BCs) depends on the postsynaptic metabotropic glutamate receptor mGluR6, located at the BC dendritic tips. Glutamate binding to mGluR6 initiates G-protein signaling that ultimately leads to BC depolarization in response to light. The mGluR6 receptor also engages in trans-synaptic interactions with presynaptic ELFN adhesion proteins. The roles of post-translational modifications in mGluR6 trafficking and function are unknown. Treatment with glycosidase enzymes PNGase F and Endo H demonstrated that both endogenous and heterologously expressed mGluR6 contain complex N-glycosylation acquired in the Golgi. Pull-down experiments with ELFN1 and ELFN2 extracellular domains revealed that these proteins interact exclusively with the complex glycosylated form of mGluR6. Mutation of the four predicted N-glycosylation sites, either singly or in combination, revealed that all four sites are glycosylated. Single mutations partially reduced, but did not abolish, surface expression in heterologous cells, while triple mutants had little or no surface expression, indicating that no single glycosylation site is necessary or sufficient for plasma membrane trafficking. Mutation at N445 severely impaired both ELFN1 and ELFN2 binding. All single mutants exhibited dendritic tip enrichment in rod BCs, as did the triple mutant with N445 as the sole N-glycosylation site, demonstrating that glycosylation at N445 is sufficient but not necessary for dendritic tip localization. The quadruple mutant was completely mislocalized. These results reveal a key role for complex N-glycosylation in regulating mGluR6 trafficking and ELFN binding, and by extension, function of the photoreceptor synapses.

Photoreceptors form glutamatergic synapses with bipolar cells (BCs) and horizontal cells in the outer plexiform layer (OPL) of the retina. Both rod and cone photoreceptors relay information to ON-type BCs, which depolarize in response to light increments. Cones also synapse with OFF-type BCs, which depolarize in response to light decrements. Metabotropic glutamate receptor 6 (mGluR6) is a G protein–coupled receptor responsible for neurotransmitter detection at dendritic tips of both rod and cone ON-BCs (1, 2, 3, 4). In the dark, tonic activation of mGluR6 results in G_αo_-dependent inhibition of the TRPM1 transduction channel by an unknown mechanism (Fig. 1*A*). Light onset deactivates mGluR6 and results in channel opening and cell depolarization (5).

**Figure 1:**
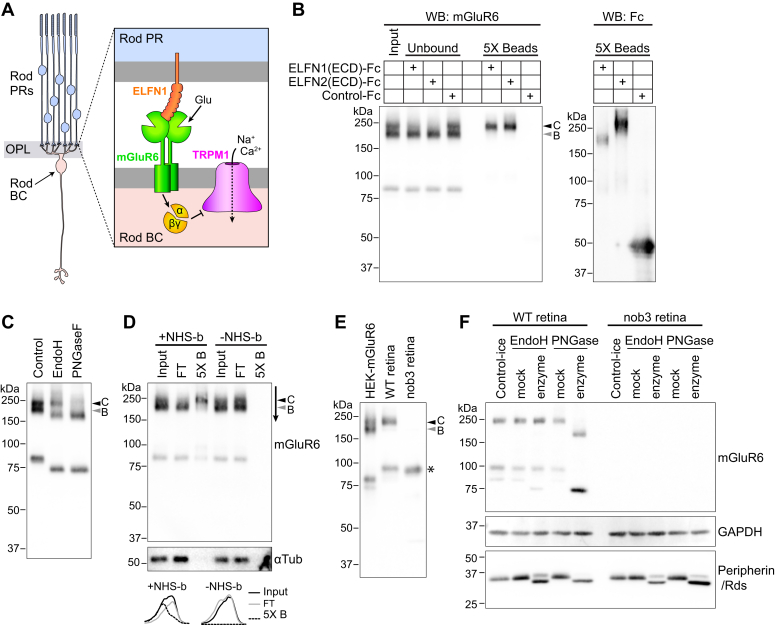
**ELFN1 and ELFN2 interact with complex N-glycosylated mGluR6.***A*, *left*: diagram of rod photoreceptors forming synapses with a BC in the OPL. *Right*: simplified diagram of the synapse showing mGluR6-mediated G-protein activation and trans-synaptic interaction with ELFN1. G_αo_ and/or G_βγ_ negatively regulate the TRPM1 ion channel. *B*, ECDs of ELFN1 or ELFN2, fused to Fc, or negative control Fc were precipitated from transfected HEK cell culture media using protein G beads. The Fc-bound beads were then incubated with solubilized lysate from HEK cells expressing mGluR6. Samples were blotted with mGluR6 mAb-312; input, flow-through, and bead-bound fractions (5× equivalent amount) are shown. Bead samples were also blotted with anti-human antibody to detect the Fc proteins. The mGluR6 upper dimer band (band C, *black arrowhead*) is present in the bead-bound fraction for both ELFN1 and ELFN2. *C*, lysate from HEK cells transfected with mGluR6 was treated with Endo H or PNGase F, and mGluR6 was detected by Western blotting with mAb-312. Bands B and C are core-glycosylated and complex-glycosylated mGluR6, respectively. *D*, HEK cells transfected with mGluR6 were treated with a cell impermeant biotinylation reagent (+NHS-b) or mock-treated (-NHS-b), and then cells were lysed and biotinylated proteins were precipitated with streptavidin beads. Input, flow-through (FT), and bead-bound fractions were blotted with mAb-312 or tubulin antibody. *Bottom*, profiles of lines drawn through dimer bands as indicated by *arrow* next to the blot. *E*, Western blot of HEK cell lysate and homogenized retina from WT CD1 or nob3 mice using mAb-312. *Asterisk* indicates endogenous immunoglobulin G heavy chains detected by anti-mouse secondary antibody. Specificity of the mGluR6 mAbs in transfected cells is shown in Fig. S1. *F*, retina lysates from WT or mGluR6-null nob3 mice were treated with Endo H or PNGase F or mock-treated. Samples were subjected to Western blotting and duplicate membranes were blotted for mGluR6 using mAb-1438, followed by mouse light-chain–specific secondary antibody (*top*) or for peripherin/Rds (*bottom*); the mGluR6 membrane was subsequently blotted for GAPDH, which was detected in a different channel (*middle*).

Trans-synaptic interactions between mGluR6 and the presynaptic leucine-rich repeat proteins ELFN1 and ELFN2 are critical for development of OPL synapses (6, 7). In adult mice, ELFN1 and ELFN2 are restricted to rod and cone synapses, respectively (6, 7). *Elfn1* KO mice exhibit malformation and malfunction of rod synapses, including loss of mGluR6, with apparently normal cone synapses (6). In contrast, ELFN1 and ELFN2 are both expressed at cone synapses during development and appear to have redundant roles; double knockout is required to ablate cone synapse function (7). ELFN proteins can also allosterically modulate mGluR6 function, suggesting additional roles in tuning synapse activity (7, 8). Similarly, ELFN1 and ELFN2 interact with and allosterically modulate other group III mGluRs—mGluR4, mGluR7, and mGluR8—affecting synapse function in the brain (8, 9, 10, 11, 12). Consistent with this, mutations in the *ELFN1* gene have been associated with neurological and psychiatric disorders (10, 13, 14, 15). With the exception of mGluR6, the group III mGluRs are typically located in presynaptic membranes, where they influence glutamate release and interact with postsynaptic ELFN proteins (10, 11, 16, 17). The photoreceptor-BC synapse is unusual in that mGluR6 serves as the postsynaptic neurotransmitter receptor, while ELFN1 and ELFN2 are located presynaptically.

The mechanisms that target mGluR6 to postsynapses in BCs are unknown, though it has been suggested that trans-synaptic interactions with ELFN proteins may be required to recruit and/or maintain mGluR6 at the synapse (6, 7, 18). mGluR6 is a class C GPCR with a large extracellular domain (ECD) that comprises a Venus flytrap ligand-binding domain (LBD) and a cysteine-rich domain (CRD) (19). Previously, we found that regions in the LBD were necessary for both ELFN1 binding and dendritic tip localization in BCs, but not plasma membrane (PM) trafficking in heterologous cells (18). The small C-terminal cytoplasmic domain was dispensable for dendritic tip localization in BCs, though deletions in this domain were reported to affect PM trafficking (18, 20, 21).

Most transmembrane proteins are modified with glycans at their ECDs, but the roles of these modifications in protein function and protein–protein interactions are diverse and poorly characterized (22). In this study, we demonstrate that Golgi-acquired complex N-linked glycosylation of mGluR6 is required for ELFN1 and ELFN2 binding. Mutation of all four glycosylated Asn residues abolished ELFN1 binding and PM trafficking in heterologous cells, as well as dendritic tip localization in BCs. The results demonstrate that N-glycosylation, and in particular complex glycosylation acquired in the Golgi, has a key role in mediating mGluR6 function.

## Results

### ELFN1 and ELFN2 preferentially interact with complex N-glycosylated mGluR6

The predicted molecular mass of the mGluR6 precursor protein is ∼95 kDa and that of the mature protein after cleavage of the N-terminal signal peptide is ∼92 kDa. In SDS-PAGE, mGluR6 from rodent retina tissue migrates as a presumptive dimer of ∼200 kDa (2, 18, 23, 24). We observed that mouse mGluR6 expressed in HEK cells by transient transfection migrated as a doublet near the expected dimer size. The doublet bands had apparent molecular masses of 199 ± 5.4 kDa and 225 ± 6.2 kDa (SD, n = 14); for clarity, we call these dimer bands B and C, respectively (Fig. 1*B*). A presumptive monomer band was also present in HEK cells, with an apparent molecular mass of 84.8 ± 1.4 kDa (SD, n = 14). ELFN1 and ELFN2, which are primarily expressed in rods and cones, respectively, were previously shown to interact with mGluR6 *via* their ECDs (6, 7). Pull-down experiments with the ECD of ELFN1 or ELFN2 fused to Fc revealed that both of these proteins bind exclusively to the mGluR6 upper dimer band (band C) (Fig. 1*B*). While band C was depleted from the unbound flow-through fraction in the ELFN-containing samples, band B was present at similar levels in input and flow-through fractions, demonstrating that the lack of binding of band B was not due to degradation of this species during the reaction. None of the mGluR6 bands were present in the negative control Fc sample.

To test the hypothesis that the different apparent sizes of bands B and C reflect a difference in glycosylation status, HEK cell lysates from cells transfected with mGluR6 were subjected to treatment with glycosidase enzymes (Fig. 1*C*). All bands were susceptible to PNGase F, indicating that the proteins were N-glycosylated. Band C was resistant to Endo H, indicating the presence of complex N-glycosylation acquired in the Golgi, while band B, as well as the monomer band, were susceptible, indicating they contain immature core glycosylation only. These findings suggest that only the complex N-glycosylated form of mGluR6 interacts with ELFN1 and ELFN2.

### Both complex and core-glycosylated mGluR6 are at the PM in HEK cells

To determine which mGluR6 species are present at the PM, intact cells were treated with a membrane impermeant biotinylation reagent, followed by cell lysis and precipitation with streptavidin beads (Fig. 1*D*). Band C was present in the biotinylated fraction and depleted from the flow-through unbound fraction, indicating that most of the complex glycosylated protein is located at the PM. Meanwhile, the Endo H–sensitive band B was not biotinylated, suggesting that this species is wholly intracellular. Surprisingly, the monomer band, which is also Endo H sensitive, was present in the biotinylated fraction. No mGluR6 was detected in controls where the biotinylated reagent was omitted, ruling out nonspecific binding to beads, and no tubulin was detected in the biotinylated fraction, indicating that the biotinylation reagent remained extracellular and was fully quenched before cell lysis. These results reveal two pools of mGluR6 at the PM in HEK cells: (1) complex glycosylated mGluR6 that migrates as an SDS-resistant dimer and (2) core-glycosylated mGluR6 that migrates as a monomer.

### Endogenous mGluR6 in retina is complex glycosylated

To compare the migration of endogenous mGluR6 with bands B and C, we separated homogenized retina from WT CD1 or mGluR6-null nob3 (25) mice along with HEK cell lysate side by side in SDS-PAGE (Fig. 1*E*). Retina mGluR6 migrated predominantly as a single band with similar mobility as band C (228 ± 5.0 kDa (SD, n = 6)), suggesting that the majority of the retinal pool is complex N-glycosylated. Consistent with this, the majority of retinal mGluR6 was Endo H–resistant and PNGase F–sensitive (Fig. 1*F*). In some experiments, Endo H–resistant monomer and faint Endo H–sensitive monomer bands could also be detected (Fig. 1*F*). Blotting the same samples for peripherin/Rds, an outer segment protein known to traffic *via* a Golgi bypass route (26), confirmed the activity of Endo H in the assay (Fig. 1*F*, bottom).

### Mutation of N-glycosylation sites in mGluR6

**Figure 2:**
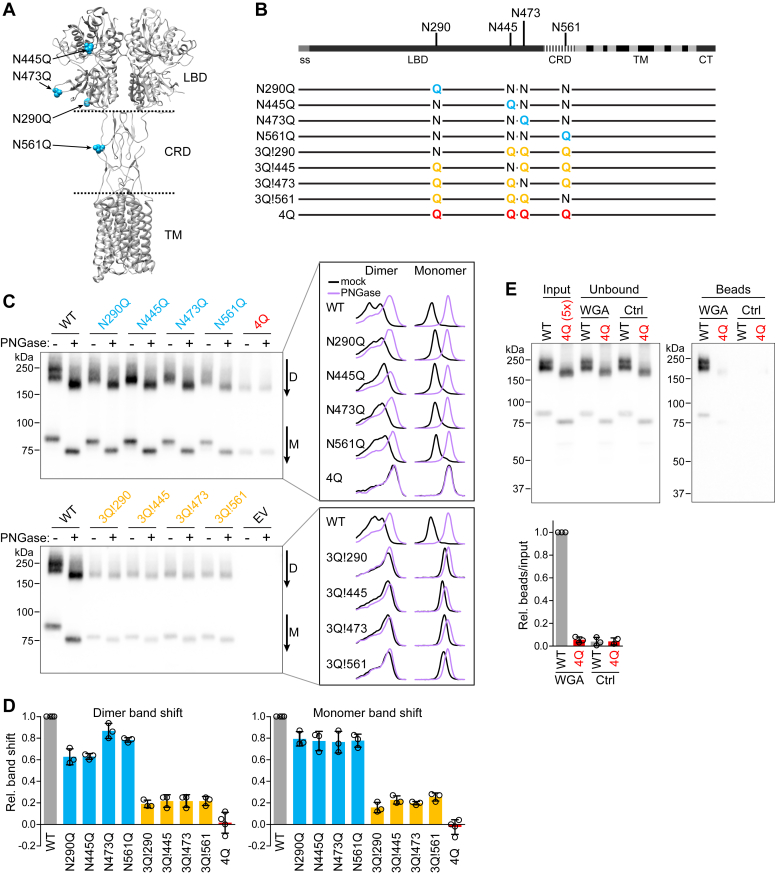
**mGluR6 glycosylation mutants.***A*, model of an mGluR4 structure (PDB 7E9H (41)) showing the approximate homologous locations of the four predicted glycosylated Asn residues in mGluR6. *B*, diagram of mGluR6 showing the position of the predicted glycosylation sites in the primary structure and mutants used in this study. *C*, lysates from HEK cells transfected with WT or mutant mGluR6 or empty vector control (EV) were subjected to PNGase F treatment, followed by blotting with mAb-312. Line profiles were drawn through dimer (*D*) and monomer (M) bands as indicated next to the blots, and example profiles are shown at *right*. *D*, mobility shifts after PNGase F treatment were quantified from the difference between the peaks of line profiles of mock- and PNGase F–treated samples. When two peaks were evident in the profile, the lowest band (right-hand peak in the profiles) was used for quantification. Points represent values from different experiments, and bars show means ± S.D. *E*, lysates from cells transfected with WT or 4Q mGluR6 were incubated with biotinylated wheat germ agglutinin (WGA) or control buffer (Ctrl), followed by precipitation with streptavidin beads. To offset the lower expression of 4Q, five times more transfected cells were used, and an equivalent amount of additional untransfected cells were pooled with the WT sample. *Bottom*, quantification of dimer bands in bead lanes relative to inputs, normalized to the WT WGA sample, for three independent experiments. CRD, cysteine rich domain; CT, C-terminal domain; LBD, ligand-binding domain; ss, signal sequence; TM, transmembrane domain.

Using NetNGlyc (27), four Asn residues in the mGluR6 ECD were predicted to be N-glycosylated (Fig. 2*A*). All four residues are conserved and also predicted to be N-glycosylated in human mGluR6. To determine the importance of glycosylation at these positions, residues were mutated to Gln, either singly or in combination (Fig. 2*B*). The dimer and monomer bands of all single and triple mutants exhibited mobility shifts when treated with PNGase F, confirming that all four positions are glycosylated. The mobility shift of triple mutants was smaller than that of single mutants, as expected. The quadruple mutant (4Q), on the other hand, exhibited no detectable change in mobility (Fig. 2*C*), suggesting that there are no additional N-glycosylation sites. Quantification of the mobility shifts relative to mock-treated controls is shown in Fig. 2*D*. Further supporting the absence of N-glycosylation in the 4Q mutant, binding to wheat germ agglutinin was severely reduced compared to WT (Fig. 2*E*). 4Q protein was still present in the unbound fraction, demonstrating that the lack of binding was not due to degradation or precipitation of the mutant.

### Mutation at N445 or N561 impairs ELFN binding

**Figure 3:**
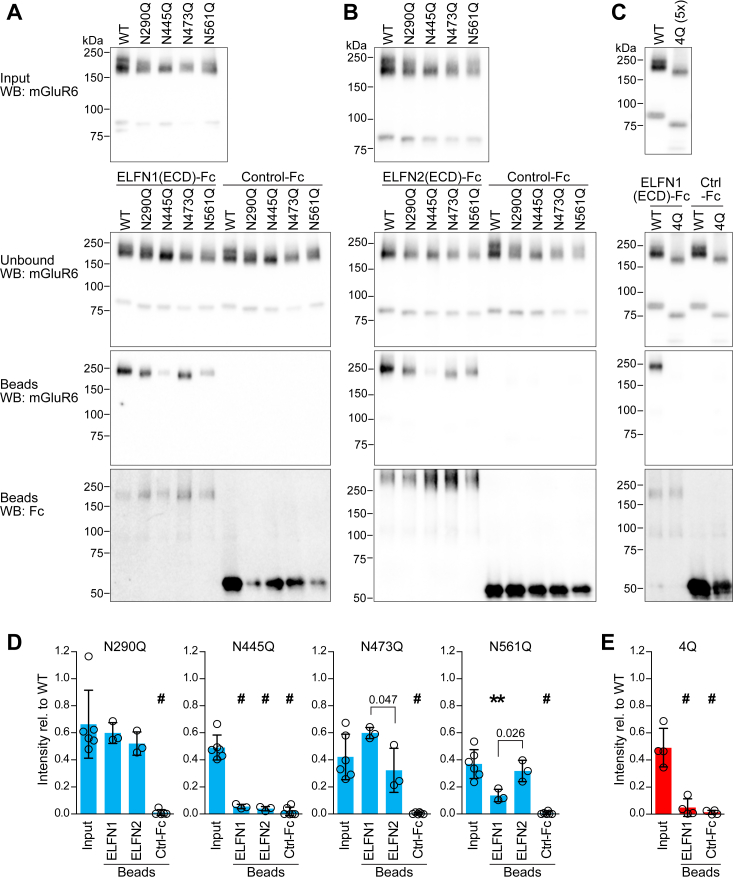
**Interaction of mGluR6 glycosylation mutants with ELFN proteins.** Pull-down assays were performed with extracellular domains of ELFN1 (*A*) or ELFN2 (*B*) and mGluR6 single mutants, as described in Figure 1. *C*, pull-down with ELFN1 and the quadruple mutant 4Q. To offset the lower expression of 4Q, five times more transfected cells were used, and an equivalent amount of additional untransfected cells were pooled with the WT sample. *D* and *E*, band intensities were quantified and normalized to the WT positive control on the same blot. For each mutant, normalized values for input and bead-bound fractions are plotted together and analyzed by one-way ANOVA with Dunnett’s post test to compare bead samples to inputs. ∗∗*p* < 0.01; #*p* < 0.001. For each mutant, ELFN1 and ELFN2 binding were also directly compared by *t* test and *p* values < 0.05 are shown. Points represent values from independent experiments, and bars show means ± SD.

To determine the effect of glycosylated residues on ELFN binding, we performed pull-down experiments with ELFN1 or ELFN2 ECDs fused to Fc (Fig. 3). The N290Q and N473Q single mutations did not considerably reduce binding to either ELFN protein when the decreased input expression levels are taken into account. In contrast, N445Q exhibited severely impaired binding to both ELNF1 and ELFN2 (Fig. 3, *A*, *B*, and *D*). Interestingly, while the N561Q mutation was deleterious for ELFN1 binding, we did not detect a significant effect on ELFN2 binding (Fig. 3*D*). Mutating all four residues resulted in severely decreased ELFN1 binding, comparable to that of the single mutant N445Q (Fig. 3, *C* and *E*). All proteins were detected in unbound fractions at similar levels as the inputs, indicating that mutants were not degraded during the binding reaction. These results suggest that glycosylation at N445 is necessary for trans-synaptic interactions with both ELFN1 and ELFN2.

### Mutation at N445 reduces endo H–resistant glycosylation

**Figure 4:**
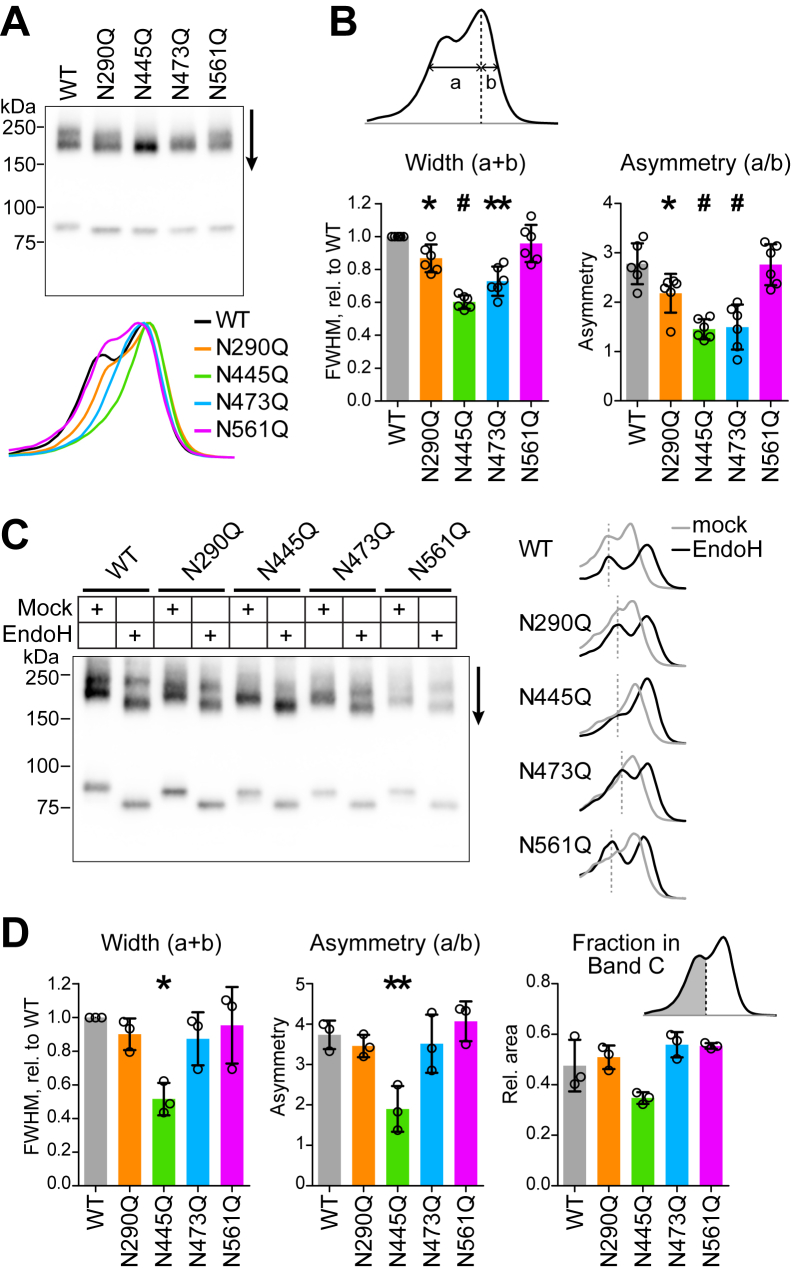
**Contribution of glycosylation sites to band C.***A*, line profiles of input samples from pull-down experiments. *B*, quantification of full-width at half maximum (FWHM, *left*) and asymmetry at half maximum (*right*) of line profiles. Asymmetry was calculated relative to band B (right hand peak). Widths are normalized to WT from the same blot and compared to 1 using one-sample *t* tests; *p* values were adjusted to correct for multiple comparisons (four). Asymmetry values were compared to WT using one-way ANOVA and Dunnett’s post test. ∗*p* < 0.05; ∗∗*p* < 0.01; and #*p* < 0.001. *C*, HEK lysates were treated with Endo H and line profiles obtained from the dimer bands. *D*, FWHM and asymmetry of profiles of the Endo H–treated lanes were calculated and analyzed as in (*B*). Because peaks were well-separated after Endo H treatment, fractional area under band C (left hand peak) was also calculated as shown in the inset, and values compared to WT with one-way ANOVA and Dunnett’s post test. Points represent values from independent experiments, and bars show means ± SD.

In the input samples of pull-down experiments (Fig. 3), we observed that the mutants appeared to contain varying amounts of band C. Line profiles through dimer bands had narrower peaks for N445Q and N473Q mutants (Fig. 4*A*). Profiles for both of these mutants had the largest reduction in full-width at half max and peak asymmetry (Fig. 4*B*), suggesting that N445 and N473 might be important for complex glycosylation. However, treatment of the mutants with Endo H revealed clear Endo H–resistant bands in all single mutants except N445Q (Fig. 4*C*). Line profiles of the Endo H–treated samples show that N473Q does in fact contain an Endo H–resistant band that is not well-resolved from the Endo H–sensitive band in the absence of glycosidase treatment (Fig. 4*D*). These results indicate that N445Q is the only mutant significantly lacking band C, which is consistent with the severe effect of N445Q on ELFN1 and ELFN2 binding (Fig. 3).

### Mutation of N-glycosylation sites reduces surface expression in HEK cells

**Figure 5:**
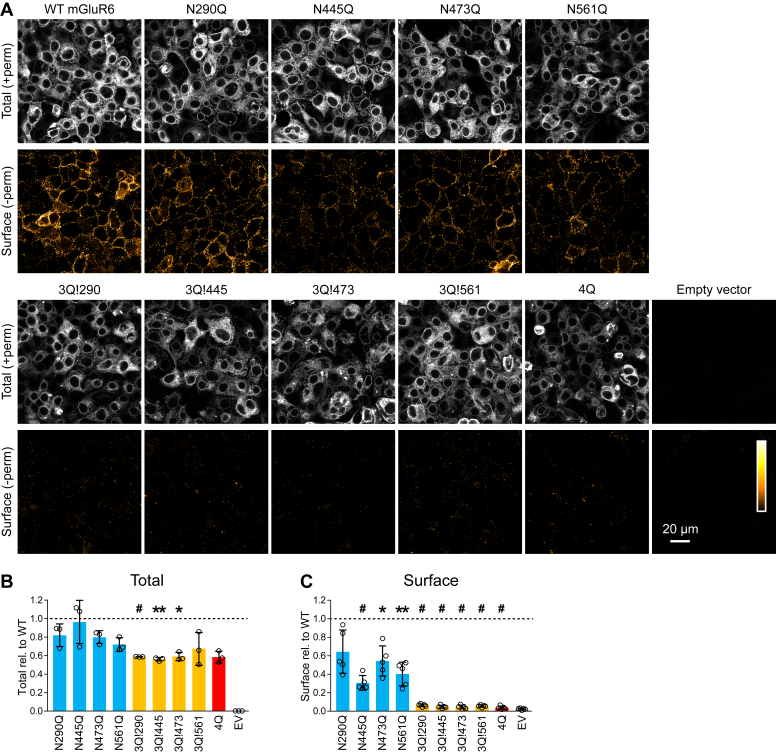
**Plasma membrane localization of glycosylation mutants in HEK cells.***A*, transfected cells were labeled with mAb-1438 in permeabilizing (*gray*) or nonpermeabilizing (*orange*) conditions. *B* and *C*, labeling intensity was quantified from images as in (*A*), and each image was normalized to the average of WT images from the same day. Mutants were compared to 1 using one-sample *t* tests, and *p* values were adjusted to correct for nine multiple comparisons: ∗*p* < 0.05; ∗∗*p* < 0.01; and #*p* < 0.001. Each point represents the mean of 3 to 6 images from an independent experiment; bars show means ± SD of independent experiments.

Immunolabeling in nonpermeabilizing conditions with an extracellular epitope antibody (mAb-1438) (18) was used to assay PM localization in HEK cells (Fig. 5, *A* and *C*). We observed that all of the single mutants, with the possible exception of N290Q, had partially reduced surface expression, with N445Q and N561Q conferring the largest defects. Triple mutants, in which only one glycosylation site remains, all had little or no surface expression, and mutating all four N-glycosylation sites entirely abolished surface expression. Parallel samples labeled in permeabilizing conditions (Fig. 5, *A* and *B*) demonstrated that total expression was reduced for triple and 4Q mutants, but none were on average below 50% of WT. These results demonstrate that while N-glycosylation is required for PM trafficking, no single site is either necessary or sufficient. In permeabilized cells, all mutants as well as WT mGluR6 largely colocalized with a coexpressed endoplasmic reticulum (ER) marker (Fig. S2).

In preparation for experiments with mGluR6-EGFP expression in retina, we also tested PM localization of WT and mutant mGluR6-EGFP in HEK cells (Fig. S3). These constructs also have the advantage of allowing visualization of surface and total protein in the same field. Surface expression detected with mAb-1438 in nonpermeabilizing conditions was consistent with that of untagged variants, with N445Q and N561Q having the most notable effects on surface expression (Fig. S3, *A* and *C*). However, total expression measured by EGFP fluorescence differed from that of the untagged mutants. N561Q-EGFP, as well as the triple and 4Q mutants, all had significantly reduced total expression, suggesting misfolding or degradation exacerbated by the EGFP fusion (Fig. S3, *A* and *B*). Interestingly, N561Q had the lowest total expression among the single mutants, and 3Q!561 had the highest expression among the triple mutants, indicating that in the context of the mGluR6-EGFP fusion, N561 may be important for protein folding.

### Mutation at N445 abolishes glutamate-induced receptor function

**Figure 6:**
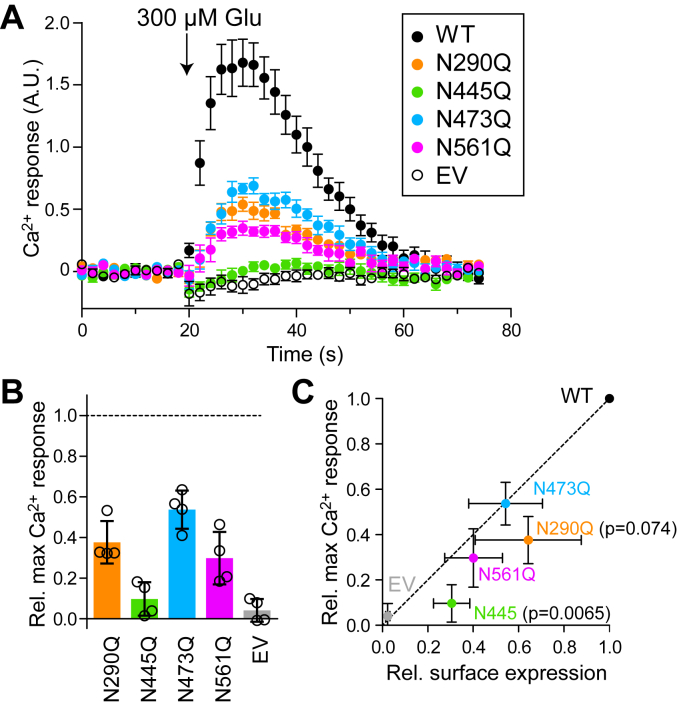
**Glutamate-induced G-protein activation of glycosylation mutants.***A*, cells cotransfected with mGluR6 and G_αqo_ chimera were loaded with fluo-4-AM calcium dye, and fluorescence was measured during addition of 300 μM glutamate. Example traces are shown; points and error bars represent mean ± SEM of five technical replicates. *B*, maximum values from three independent experiments are shown, normalized to WT from the same plate. Bars represent means ± S.D. *C*, mean maximal Ca^2+^ responses are shown plotted against mean surface expression (from Fig. 5). Error bars represent SD. For each mutant, Ca^2+^ responses were compared to surface expression values by *t* test; *p* values <0.1 are shown.

Because the single mutants had easily detectable surface expression (Fig. 5), we tested receptor function in HEK cells using a Ca^2+^ mobilization assay (Fig. 6). Cells were cotransfected with the chimeric G_αqo_ to enable G_o_-coupled mGluR6 to engage the G_q_-mediated Ca^2+^ mobilization pathway (28, 29). All single mutants except N445Q exhibited functional G-protein activation in response to 300 μM L-glutamate. N445Q, in contrast, did not mediate any detectable Ca^2+^ response (Fig. 6, *A* and *B*). To assess receptor function in light of the reduced surface expression of the mutants, we compared the relative maximal activity to relative surface expression (from Fig. 5) of each mutant (Fig. 6*C*). The relative activity of N445Q was significantly lower than its relative surface expression (Fig. 6*C*), suggesting that this mutation confers functional defects beyond the expected reduction proportional to the reduction in surface expression. The N290Q mutation also appeared to have a disproportionate effect on G-protein activation (Fig. 6*C*), though it did not reach statistical significance at a threshold of 0.05.

### N-glycosylation is required for dendritic tip localization in BCs

**Figure 7:**
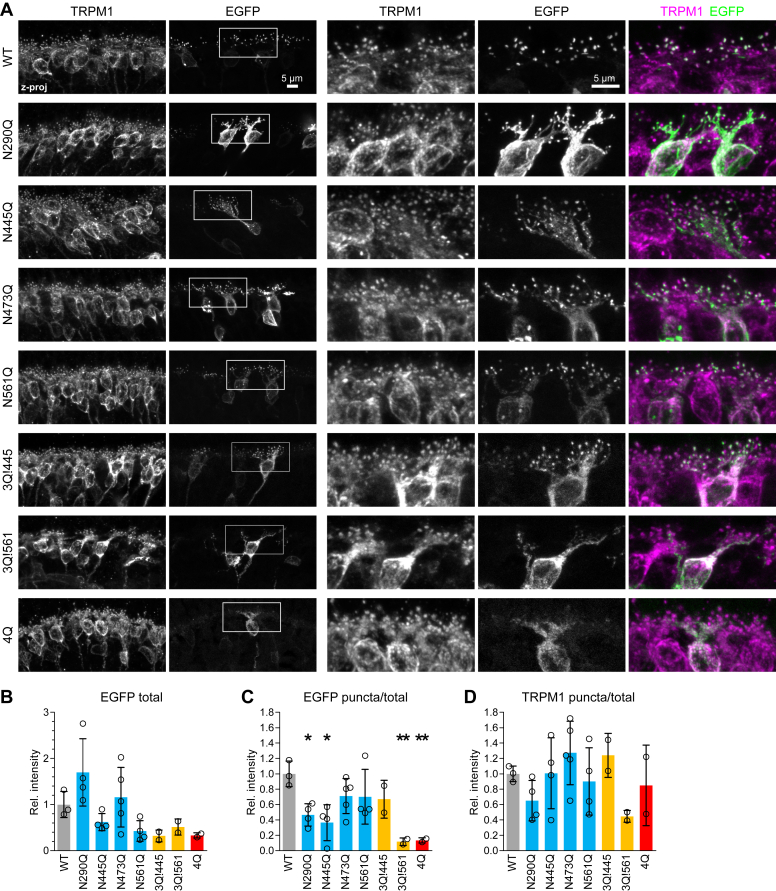
**Localization of glycosylation mutants in mouse retina.***A*, WT or mutant mGluR6-EGFP (*green*) was expressed in WT CD1 mouse retinal BCs by subretinal injection and electroporation. Retina sections were costained with TRPM1 mAb-545H5 (*magenta*). Images on the *right* are magnified views of the *boxed regions*. Images were processed to highlight the localization of each mutant and intensities should not be compared. *B-D*, intensity of OPL puncta in EGFP and TRPM1 channels, as well as total EGFP, were quantified as described in the Experimental procedures. Values were compared to WT using one-way ANOVA and Dunnett’s post test. ∗*p* < 0.05 and ∗∗*p* < 0.01. Each point represents the mean of at least three (median n = 5) images from a different animal, and error bars show means ± SD. of biological replicates.

To examine the localization of mutants in BCs, WT or mutant mGluR6-EGFP constructs were introduced in WT mouse retinas by subretinal injection and electroporation (30) at postnatal day 0. The 200 bp critical region of the *Grm6* promoter was used to restrict expression to ON-BCs (31). Four to six weeks postinjection, retina sections were labeled with TRPM1 antibody and OPL puncta were quantified in the TRPM1 and EGFP channels (Fig. 7). WT mGluR6-EGFP exhibited normal dendritic tip localization, as we reported previously (18, 32). All single mutants were capable of enrichment in TRPM1-containing puncta in the OPL (Fig. 7, *A*–*C*), indicating that no single glycosylation site is absolutely necessary. Surprisingly, the 3Q!445 triple mutant was also readily detectable in OPL puncta, indicating that glycosylation at N445 is sufficient for dendritic tip enrichment. In contrast, the 3Q!561 triple mutant and 4Q quadruple mutant were completely mislocalized, with no detectable dendritic tip enrichment (Fig. 7, *A* and *C*).

Total intensity in the EGFP channel (Fig. 7*B*) demonstrates that analyzed images contained EGFP-positive cells, though the number of positive cells and expression levels varied. Although total expression varied between mutants, none were significantly different from WT. We did not detect strong correlations between total EGFP and the fraction in OPL puncta, suggesting that expression level is not a major determinant of dendritic tip trafficking, and that the puncta detection method is not sensitive to EGFP intensity (Fig. S4). Quantification of puncta relative to total intensity in the TRPM1 channel was similar for most mutants (Fig. 7*D*) and demonstrates that dendritic tips were present in the analyzed images. Similar results were observed for puncta-forming mutants expressed in nob3 retina, indicating that dendritic tip trafficking is not mediated by dimerization with endogenous mGluR6 (Fig. S5).

### Removal of N-glycosylation affects SDS-resistant mGluR6 dimer

**Figure 8:**
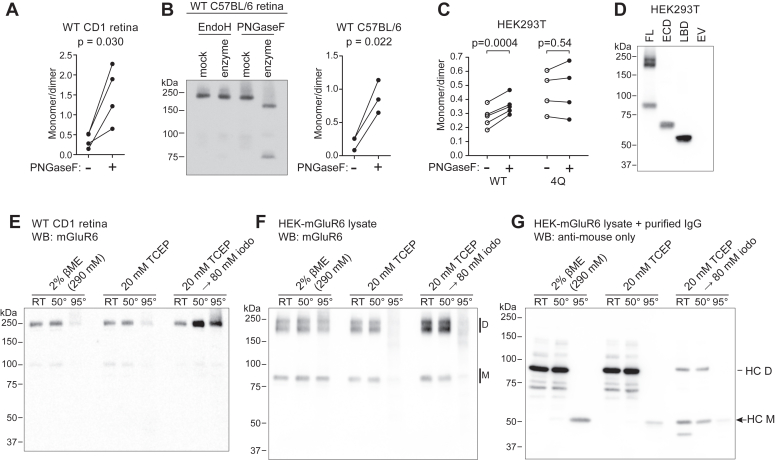
**Abundance of mGluR6 monomer band is affected by glycosylation status.***A*–*C*, PNGase treatment reduces abundance of SDS-resistant dimers. *A*, quantification of WT CD1 retina lysates treated with PNGase F, as shown in Figure 1*F*. Intensities of monomer bands divided by dimer bands from the same lane are shown, and PNGase F–treated samples were compared to mock-treated samples by paired *t* test. *B*, lysates from WT C57BL/6 retina were treated similarly with PNGase F or mock-treated, detected by Western blot with mGluR6 mAb-1438, and monomer/dimer intensities were compared by paired *t* test. *C*, monomer/dimer band intensities were quantified from WT and 4Q mutant samples treated with PNGase F, as shown in Figure 2*C*. PNGase F–treated samples were compared to the associated mock-treated controls by paired *t* tests. *D*, Western blot of HEK cell lysates expressing full-length mGluR6 (FL), ECD or LBD fragments, or empty vector (EV) control. *E* and *F*, neither heating nor cysteine alkylation improves detection of monomers in retina (*E*) or HEK (*F*) samples, using mGluR6 mAb-312. *G*, the same HEK lysate from (*E*) was spiked with a purified mouse IgG1 and blotted with anti-mouse antibody to demonstrate recovery of heavy chain monomers (HC M, *arrow*) from heavy-chain dimers (HC D) in the same conditions. Retina samples in (*B* and *E*) were blotted with mGluR6 mAb, followed by light-chain–specific secondary antibody.

Following PNGase treatment of retina lysates, we observed a large increase in the intensity of a ∼75 kDa band (Figs. 1*F* and 8*A*). To confirm that this band represents mGluR6 and not endogenous immunoglobulin G (IgG) heavy chain detected by the anti-mouse secondary antibody (Fig. 1*E*) (18, 33), we included parallel samples from mGluR6-null nob3 mouse retina (25) and used a light-chain–specific secondary antibody (Fig. 1*F*). Similar results were observed with WT C57BL/6 mice (Fig. 8*B*). To determine whether this effect could also be detected in mGluR6 expressed in HEK cells, we reanalyzed blots from PNGase F experiments including those shown in Fig. 2*C*. Compared to mock-treated controls, there was a small but significant increase in the intensity of the monomer band relative to the ensemble of dimer bands (Fig. 8*C*). In contrast, the ratio of monomer to dimer of the 4Q mutant was not significantly different after PNGase treatment (Fig. 8*C*). Together, these results suggest that removal of N-glycosylation reduces the stability or formation of the SDS-resistant dimer band.

The nature of mGluR6 dimers observed in the denaturing and reducing conditions of SDS-PAGE is unknown. However, the SDS-resistant dimer appears to be mediated by the transmembrane domain (TM), since neither the LBD nor ECD (LBD + CRD) fragments migrate as dimers (Fig. 8*D*). In our standard protocol, samples are loaded without heating. For both retina and HEK samples, incubation at higher temperature failed to increase abundance of the monomer species and the 95 °C treatment typically led to reduction of total mGluR6 detected (Fig. 8, *E* and *F*). Alkylation of cysteine sulfhydryl groups with iodoacetamide improved detection of presumptive dimer bands, but failed to yield additional monomers (Fig. 8, *E* and *F*), while IgG heavy chain monomers were detected without heating in HEK lysate spiked with a purified mouse antibody (Fig. 8*G*).

## Discussion

In this study, we demonstrate that mGluR6 is modified with complex N-glycosylation and that this modification is required for binding to ELFN1 and ELFN2. WT mGluR6 with only immature core N-glycosylation failed to interact with ELFN proteins (Fig. 1), as did mGluR6 with Asn→Gln mutations at all four N-glycosylation sites (Fig. 3). Our results are consistent with the reported N-glycan–dependent interaction between ELFN1 and mGluR7 (34), a presynaptic group III mGluR that interacts with postsynaptic ELFN1 at synapses in the hippocampus (10). We observed a similar requirement for mGluR6 N-glycosylation, and in addition, we identified a novel specific requirement for mature glycosylation acquired in the Golgi. We also demonstrate a role for N-glycosylation in mGluR6 dendritic tip localization *in vivo* in mouse retina. ELFN1 and ELFN2 can interact with all group III mGluRs and allosterically modulate G-protein activation (7, 8, 11), suggesting broad implications for the role of mGluR glycosylation at glutamatergic synapses in the central nervous system.

In the retina, the cues specifying synapse formation between correct presynaptic and postsynaptic partners are not well understood. Expression of ELFN1 in rods is required for formation of rod synapses, while it appears that ELFN1 and ELFN2 function at different times in cones during formation of cone synapses (6, 7). Both rod and cone ON BCs express mGluR6 at their dendritic tips (4), and BC subtype-specific postsynaptic proteins that interact with ELFN proteins have not yet been identified. The N445Q mutation severely reduced mGluR6 binding of both ELFN1 and ELFN2, suggesting that glycosylation at this position is an important determinant of ELFN binding (Fig. 3). On the other hand, the N445Q mutant also had reduced Endo H–resistant glycosylation and PM trafficking (Figure 4, Figure 5, and S1), suggesting the alternative explanation that the N445Q mutation might cause some misfolding that hinders exit from the ER, thereby reducing Golgi trafficking and maturation of glycans.

In contrast to N445, which is located in the upper lobe of the LBD, mutation at N561, located in the CRD, appeared to have a larger effect on ELFN1 binding than ELFN2 binding. These results may reflect differences in the binding sites for ELFN1 and ELFN2. The ECDs of murine ELFN1 and ELFN2 share only 51% sequence identity, and it would therefore not be surprising if the mGluR6 interactions have also diverged. An interesting possibility is that different BC types might synthesize mGluR6 with subtle variations in N-glycan structure that differentially affect the interactions with ELFN1 and ELFN2 and thereby affect synapse wiring during development. Indeed, N-glycan microheterogeneity can affect protein–protein and protein–small molecule interactions and can be regulated in a cell-type specific manner (22, 35, 36, 37, 38).

All of the single mutants exhibited some degree of dendritic tip enrichment in rod BCs (Fig. 7), suggesting that no particular glycosylation site is necessary for synaptic localization. Even N445Q and N561Q, which exhibited significant defects in ELFN1 binding, still had clear dendritic tip enrichment. However, at the resolution of confocal microscopy, PM localization cannot be distinguished from intracellular membranes within the dendritic tips. Though subcellular compartments within the dendritic tips have not been reported, it is possible that there are vesicles, including recycling endosomes, and perhaps other compartments as well. It is clear that dendritic tip targeting and/or trapping of the single mutants is at least partially intact, but it remains to be determined whether the mutants are localized in the postsynaptic PM or in nearby intracellular membranes.

The triple mutant with N445 as the sole N-glycosylation site was also capable of dendritic tip enrichment in ON-BCs, while the triple mutant with only N561 remaining was severely mislocalized, demonstrating that while glycosylation at N445 is sufficient for dendritic tip enrichment, glycosylation at N561 is not. In our previous work with mGluR6 N-terminal deletion mutants, we observed uncoupling of secretory trafficking from synaptic localization; some mutants had robust surface expression in HEK cells, but failed to interact with ELFN1 and failed to localize to dendritic tips in ON-BCs (18). This, along with the mislocalization of mGluR6 in *Elfn1* KO mice (6), led to the speculation that diffusional trapping *via* trans-synaptic interactions with ELFN1 is necessary for mGluR6 synaptic enrichment. However, we observed that the triple mutant with the N445 glycosylation site remaining was capable of dendritic tip enrichment in rod BCs, despite almost no detectable PM trafficking in HEK cells. This suggests that either BCs are more permissive for secretory trafficking of this mutant to the PM or that trans-synaptic interactions are not necessary for dendritic tip enrichment.

In BCs, the trafficking routes of postsynaptic membrane proteins are poorly understood, and the locations of Golgi compartment usage and PM insertion are unknown. In our previous work we showed that, in certain labeling conditions, a somatic pool of endogenous mGluR6 can be detected in intracellular membranes partially colocalizing with the ER (18). However, glycosidase treatments showed that almost all of the mGluR6 in retina is complex glycosylated (Fig. 1). To reconcile these two results, the somatic intracellular pool must either be a small fraction of the total mGluR6 or comprise complex glycosylated mGluR6 located in post-Golgi vesicles despite appearing to colocalize with the ER. Further studies are needed to identify secretory compartments and trafficking routes of mGluR6 in BCs, as well as the specific location of trafficking-defective mutants.

Like other mGluRs (39, 40, 41, 42, 43), mGluR6 has been reported to form dimers (21), though no structures of mGluR6 have yet been reported. Single particle cryo-EM structures of full-length mGluR2, mGluR3, mGluR4, mGluR5, and mGluR7 (40, 41, 43, 44, 45) revealed that in most cases, the dimer interface in the inactive state is formed primarily by the LBD, whereas in the active state the CRD and TM may also associate. In SDS-PAGE, the migration of the major mGluR6 bands is consistent with dimers. However, formation of spurious multimers of membrane proteins observed by SDS-PAGE is common, and the mGluR6 dimer bands may not necessarily reflect physiological dimers. The observation that isolated LBD and ECD domains migrate as monomers suggests that formation of SDS-resistant dimers is mediated by the TM domain. Nevertheless, the presence of complex glycosylation predominantly in the SDS-resistant dimer suggests that glycosylation plays a role in dimer formation and/or stability. Consistent with this, PNGase treatment was found to increase the abundance of the monomer band, both in retina and in HEK cells, and this effect was negated in the 4Q mutant (Fig. 8). These results could also reflect a role for mGluR6 dimer formation in forward trafficking from the ER to the Golgi. The observation that the core-glycosylated monomer band, but not the core-glycosylated dimer band, may be present at the PM (Fig. 1), also suggests a link between dimer formation and sorting. Furthermore, the protein in the dimer bands is not converted into monomers by heating or reducing (Fig. 8), suggesting that the minor monomer bands observed in SDS-PAGE of WT mGluR6 are not derived from dissociation of dimers.

In Ca^2+^ mobilization assays, the N445Q single mutant had defects in glutamate-induced G-protein activation that were disproportionate to its reduced surface expression (Fig. 6). The results suggest that this amino acid mutation and/or loss of glycosylation at this site may impact conformational changes that translate ligand binding into G-protein activation. Though we did not measure ligand binding, in the mGluR4 structure (41) (Fig. 2) none of the homologous positions to mGluR6 N-glycosylation sites are within 20 Å of the orthosteric binding site. However, N-glycans can be quite large, and furthermore there may be secondary effects on protein structure that effect the binding site, so a defect in glutamate binding is also possible.

Our results reveal a critical requirement for mGluR6 complex N-glycosylation, and residue N445 in particular, in trans-synaptic partner interaction. Other trans-synaptic interactions involving leucine-rich-repeat transmembrane neuronal proteins were previously shown to be dependent on heparan-sulfate modification of binding partners (46), including at photoreceptor-BC synapses (32). These findings highlight the importance of glycans in the synaptic cleft. Given the heterogeneity inherent in nontemplate-driven post-translational modifications (47), they also highlight the ability of cells to use glycans to fine-tune cell-surface interactions, including trans-synaptic interactions.

## Experimental procedures

### DNA constructs

For expression in HEK cells, pCDNA3.1 with untagged mouse mGluR6 (NP_775548.2) and mGluR6-GGGSGGG-EGFP were as described previously (18). Single glycosylation mutants were made in the untagged constructs by site-directed mutagenesis, and triple and 4Q mutants were made by overlap extension PCR using a combination of WT and mutant templates and mutagenesis primers. Mutant versions of pCDNA3.1 mGluR6-GGGSGGG-EGFP were made by overlap extension PCR using untagged mutants as templates and cloned into pCDNA3.1. Mutant mGluR6-GGGSGGG-EGFP ORFs were subcloned into pGrm6P (18, 48), which contains the *Grm6* promoter 200-bp critical region and SV40 enhancer for expression in ON-BCs (31). Untagged full-length mGluR6 constructs were used for all experiments unless indicated otherwise. Isolated domains of mGluR6—C terminally Myc-tagged LBD (aa 1–497) and ECD (LBD + CRD, aa 1–578)—were cloned in pCDNA3.1. Mouse ELFN1 cDNA was from Dharmacon (Clone ID: 6811341), and mouse ELFN2 cDNA was obtained from Horizon Discovery (Clone ID: 5706857). ECDs of ELFN1 (aa 1–418) and ELFN2 (aa 1–397) were fused to human Fc (from Addgene plasmid #59313, a gift from Peter Scheiffele and Tito Serafini (49)) with a GGGAAA linker in pCDNA3.1. To construct a negative control Fc, the influenza hemagglutinin signal sequence (50), followed by a flag tag, was fused to human Fc. The mEmerald-Sec61-C-18 plasmid was a gift from Michael Davidson (Addgene plasmid #54249). pCDNA3.1 with chimeric G_αqo_ was previously described (51).

### Cells and transfections

HEK 293T cells were maintained in Dulbecco's modified Eagle medium with 10% fetal bovine serum (Corning), without antibiotics, at 37 °C in a 5% CO_2_ atmosphere. Cells seeded in 24-well plates were transfected with 0.6 ug DNA and 1 μl Lipofectamine 2000 (Invitrogen) according to the manufacturer instructions. For immunofluorescence microscopy (IF), cells were seeded on poly-D-lysine coated coverslips in 24-well plates.

### Primary antibodies

*mGluR6*: mAbs 312 and 1438 were previously described and validated using nob3 retina tissue (18, 24) and used at 1 μg/ml for Western blotting and 3 μg/ml for IF in HEK cells. *TRPM1*: mAb 545H5 was previously described and validated using *Trpm1* KO retina tissue (24, 33) and used for retina IF at 8 μg/ml. TRPM1 and mGluR6 mAbs were purified from hybridoma media containing Iscove′s Modified Dulbecco′s medium supplemented with 10% ultralow IgG serum (Sigma), 100 U/ml penicillin, and 100 μg/ml streptomycin, using a column packed with protein G Sepharose Fast Flow (GE Healthcare) as described (18). For some experiments, 1438 hybridomas were first adapted to low serum media, and antibodies were produced from culture media containing 90% Cell mAb Medium (Gibco), 8.5% Iscove′s Modified Dulbecco′s medium, 1.5% ultralow IgG serum (Avantor Seradigm), 100 U/ml penicillin, and 100 μg/ml streptomycin. Peripherin/Rds polyclonal rabbit antibody (Proteintech #18109-1-AP) was diluted to 0.7 μg/ml for Western blotting. Fc fusion proteins were detected in western blots with goat anti-human IgG Fc conjugated to DyLight 680 (Thermo Fisher Scientific #SA510138, 0.25 μg/ml).

### Glycosidase treatments

Approximately 40 to 48 h posttransfection, HEK cells were washed in PBS and resuspended in lysis buffer (PBS supplemented with 1% Triton X-100, 50 mM NaCl, and 1× or 1.5× Complete EDTA-free protease inhibitors [Roche]). After incubating on ice for 15 min, samples were centrifuged at 11200*g* for 10 min at 4 °C, and supernatants were used in glycosidase reactions. For experiments with retina, whole retinas were collected in PBS, washed three times in PBS, and homogenized by pipetting up down in 150 μl/retina PBS supplemented with Complete EDTA-free protease inhibitors (Roche), followed by ∼100 passes through a 23 G needle and ∼40 passes through a 26 G needle. Glycosidase enzymes and related reagents were from Qiagen. Nine volumes of HEK cell lysate or homogenized retina were mixed with one volume of 10× glycoprotein denaturing buffer (5% SDS, 400 mM DTT), and incubated at room temperature for 10 min. For control reactions, 30 μl denatured lysate were mixed with 10 μl PBS and kept on ice. For PNGase reactions, 30 μl denatured lysate were mixed with 4 ul Glycobuffer 2, 4 μl 10% NP40, 1 μl mock buffer (50 mM NaCl, 20 mM Tris–HCl, 5 mM EDTA, 50% glycerol, pH 7.5), and 1 μl PNGase F; for Endo H reactions, 30 μl denatured lysate were mixed with 4 μl Glycobuffer 3, 4 μl PBS, and 2 μl Endo H; mock reactions were assembled with mock buffer in place of enzymes. For experiments with C57BL/6 mice (Fig. 8*B*), retinas were homogenized in ∼70 μl/retina cracking buffer (20 mM Tris, 300 mM sucrose, 15 mM EDTA, 2 mM MgCl_2_, pH 8.1, with 2× Roche protease inhibitors) and 2 μl PNGase were used. Mock and enzyme reactions were incubated at 37 °C for one h, followed immediately by SDS-PAGE and Western blotting with mGluR6 mAb 312 or 1438.

### ELFN pull-down experiments

Pull-down experiments were performed essentially as described (18). Wells to be transfected with Fc fusions were washed once with PBS and placed in Dulbecco's modified Eagle medium containing 10% ultralow IgG fetal bovine serum (Avantor Seradigm), and cells were transfected with 0.6 μg ELFN1(ECD)-Fc, ELFN2(ECD)-Fc, or negative control HAss-flag-Fc in pcDNA 3.1. In most experiments, additional wells were transfected with ELFN1 and ELNF2, in a 4:1 or 2:1 ratio with the Fc control, to compensate for the superior expression and/or secretion of the Fc control. The next day, new cells were transfected with 0.6 μg of pcDNA 3.1 with WT or mutant untagged mGluR6. For the mGluR6 4Q mutant, five wells were used for each WT well to equalize total expression. When different numbers of wells were used for different constructs, untransfected wells were included to equalize the total number of cells.

Approximately 40 to 46 h after transfection, media was removed from Fc transfected wells and centrifuged to remove cells. After adding 1/20 volume 1 M Tris pH 7.4 and a dash of solid PMSF to the supernatant, protein G Plus agarose beads (Calbiochem) were added (20 μl bead slurry per reaction), and samples were incubated at room temperature with end-over-end mixing for 2 h, followed by 3 washes in PBS. During the incubation, cells transfected with mGluR6 were harvested, washed with PBS, and resuspended in lysis buffer as above. After lysing on ice for 15 min, samples were centrifuged at 11200*g* for 10 min at 4 °C. A portion of the supernatant was saved as input and the remainder was divided between Fc bead samples and incubated at 4 °C for 90 min with end-over-end mixing. Beads were then centrifuged and supernatant (flow-through) was saved, followed by four washes in wash buffer (PBS with 1% Triton X-100, 50 mM supplemental NaCl, and a dash of PMSF). Input lysate, flow-through, and 5× equivalent bead samples were resolved by SDS-PAGE, transferred to nitrocellulose, and blotted with mGluR6 mAb-312.

### Lectin pull-down experiments

Biotinylated wheat-germ agglutinin (Sigma #L5142) was reconstituted to 5 mg/ml in 10 mM Hepes, 150 mM NaCl, 0.1 mM CaCl_2_, pH 7.5. HEK cells were transfected with mGluR6 4Q (5–6 wells) or WT (1 well). Wells containing untransfected cells or empty vector-transfected cells were pooled with WT to equalize total cells. HEK cell lysates were prepared as for ELFN pull-down experiments, except that 0.1% SDS was included in the lysis buffer. Thirty micrograms of lectin was added to 300 μl cell lysate, and these samples, along with mock controls without lectin, were incubated overnight at 4° with end-over-end mixing. Fifteen microliters of streptavidin-agarose bead slurry (Thermo Fisher Scientific) were added to each reaction, followed by 2 more hours of incubation at 4° with end-over-end mixing, and then washed as for ELFN pull-down experiments, except that 0.1% SDS was included in the wash buffer.

### Surface biotinylation

Two wells in a 24-well plate were transfected with pCDNA3.1 mGluR6 (untagged). Approximately 40 to 46 h posttransfection, media was removed and cells were harvested in PBS, then washed twice with PBS-8 (PBS pH 8), resuspended in PBS-8, and divided in half. Cells were incubated with 10 mM EZlink-Sulfo-NHS-SS-biotin (Thermo Fisher Scientific) dissolved in PBS immediately before use or mock-treated with PBS. Reactions were quenched with six volumes of PBS-8 with 100 mM glycine, and cells were pelleted and washed twice in quenching solution. Cells were lysed in 50 mM Tris pH 7.4, 200 mM NaCl, 1% Triton X-100, 1× Complete EDTA-free protease inhibitors (Roche), incubated on ice for 15 min, then centrifuged at 11200*g* for 10 min at 4 °C. Supernatants were mixed with streptavidin agarose beads (Thermo Fisher Scientific), allowed to bind for ∼90 min, then washed four times with wash buffer as for protein G pull-down experiments.

### Western blotting

For SDS-PAGE, samples were mixed with 4 volumes of 5× sample buffer containing 250 mM Tris pH 6.8, 10% SDS, 50% glycerol, and 10% β-mercaptoethanol. Samples were loaded onto Tris-glycine gels without heating unless indicated otherwise. After transferring to nitrocellulose, membranes were blocked in 5% milk in 50 mM Tris-HCl, 150 mM NaCl, 0.1% Tween-20, pH 8.4, incubated with primary antibody in blocking solution overnight, followed by goat anti-mouse conjugated to horseradish peroxidase (HRP) (Jackson Immunoresearch, 0.16 μg/ml), goat anti-rabbit conjugated to HRP (Proteintech, 1:5000), or rabbit anti-mouse (kappa light chain) conjugated to HRP (Proteintech, 1:10000) for two h. SuperSignal Pico (Thermo Fisher Scientific) chemiluminescent substrate was added and blots were imaged with an Azure 500 digital imager. Imaging settings were adjusted to avoid saturation.

For experiments testing different disruption conditions (Fig. 8), HEK lysates or retina homogenates as described above were incubated with 2% SDS and the indicated reducing agent and temperature for 10 min. Some samples were then mixed with 1/9 volume of iodoacetamide prepared fresh in PBS and incubated for 30 min at room temperature in the dark, while the remaining samples were kept on ice. Standard 5× sample buffer as described above was then added to each sample before loading the gel. As a control, parallel reactions were performed with HEK cell lysate supplemented with 50 μg/ml purified mouse IgG1 antibody (anti-TRPM1 mAb 1109F9 (24)).

Molecular masses were estimated from blots by fitting a line to a standard curve constructed from the measured migration of the 250, 150, 100, and 75 kDa marker bands plotted against the log of the molecular weight. For quantification of band shifts following glycosidase treatments, intensity profiles were obtained in ImageJ (National Institutes of Health) using 5px wide vertical lines and the “plot profile” tool. Profile maxima were manually identified from the list of intensities, and the difference between maxima positions in control/mock and enzyme-treated lanes, normalized to that of WT on the same blot, are reported. For profiles with two maxima, such as the doublet dimer bands, the bottom-most band (right hand peak) was used. For quantification of band intensity, rectangular regions of interest (ROIs) were drawn around bands in ImageJ, and background boxes of identical size were placed immediately below each band. Raw integrated density was measured and background boxes were subtracted.

### Surface expression assay

HEK 293T cells growing on poly-D-lysine coated coverslips were transfected with 0.6 μg of pCDNA3.1 with either untagged mGluR6 or mGluR6-EGFP. Approximately 40 to 46 h posttransfection, media was removed from the wells and the cells were fixed in 2% paraformaldehyde in PBS for 10 min. After three washes in PBS, the cells were blocked for 15 min in either PBSA (PBS + 1% bovine serum albumin) for labeling in nonpermeabilizing conditions or PBSAT (PBSA + 0.1% Triton X-100) for labeling in permeabilizing conditions. Cells were labeled for 1 h at room temperature with mAb-1438 (3 μg/ml) diluted in either PBSA or PBSAT. Wells were then washed three times in PBS and labeled at room temperature for 30 min with donkey anti-mouse IgG conjugated to Alexa 555 (Invitrogen) diluted to 2 μg/ml in PBSA or PBSAT. Following 3 washes in PBS, coverslips were mounted with ProLong Diamond antifade mountant (Invitrogen).

Coverslips were imaged using a Zeiss LSM880 confocal microscope with a 63× oil immersion objective. Alexa 555 and EGFP were imaged using 561 nm and 488 nm lasers, respectively; 4 to 6 images (1024 × 1024 pixels, 132 nm/pixel) were acquired from each coverslip. For each experiment, WT and mutant samples were imaged together using identical settings. Images in figures are shown with identical level adjustments. Raw images were processed with a custom script in Mathematica v13 (Wolfram); as an approximate background correction, images were thresholded to set values ≤2% to zero, then the mean intensity of each image was determined. All values were divided by the average of WT images from the same experiment.

### Ca^2+^ mobilization experiments

Clear 96-well plates were coated with poly-D-lysine and seeded with 30,000 to 40,000 HEK 293T cells per well. The next day, cells were cotransfected with 100 ng of pcDNA3.1-mGluR6 untagged WT or mutant constructs and 150 ng of chimeric G_αqo_ using 0.5 μl/well Lipofectamine 2000 (Invitrogen) according to the manufacturer’s instructions. Approximately, 42 h to 46 h post transfection, Ca^2+^ mobilization assays were carried out as follows. Cells were first washed with 100 μl of KRH (120 mM NaCl, 4.7 mM KCl, 2.2 mM CaCl_2_, 10 mM Hepes, 1.2 mM KH_2_PO_4_, 1.2 mM MgSO_4_, 1.8 g/L glucose, pH 7.4, supplemented with 1 mM probenecid). Cells were then incubated with 50 μl of dye loading solution (2.7 μM Fluo-4 AM (Invitrogen) in KRH with 0.06% Pluronic F-127 (Biotium)) for 1 h in the dark at room temperature. Cells were washed again with KRH and then left in 120 μl of KRH for the assay. A 4× L-glutamate solution (1.2 M) was made in KRH, and both the cell plate and drug solution were preincubated for 10 min in a SpectraMax i3x plate reader (Molecular Devices) preheated to 37 °C. Measurements were acquired every 2 s (Ex/Em 488/520) for 75 s per well with 40 μl of the drug solution added after 20 s, such that the final concentration of glutamate was 300 μM. Traces from 4 to 6 technical replicates were averaged, and the maximum value was divided by the maximum of WT from the same plate.

### Animals

Protocols were approved by the Dalhousie University Committee on Laboratory Animals, and all procedures were performed in accordance with regulations established by the Canadian Council on Animal Care. Work done at Baylor College of Medicine was approved by the Baylor Animal Care and Use Committee. WT CD1 mice (CD1-Elite #482) were from Charles River Laboratories. *Grm6*^*nob3*^ mice (25), referred to here as nob3, were previously obtained from Jackson Laboratory (#016883) and back-crossed to CD1 (Charles River #022) for five generations (18). C57BL/6 mice were from the Baylor College of Medicine Center for Comparative Medicine, and absence of the *Crb1*^*rd8*^ allele was confirmed by PCR amplifying and sequencing the relevant fragment of genomic DNA (52). All experiments were performed with CD1 mice unless indicated otherwise.

### Subretinal injection and electroporation

Retinal neurons were transduced with plasmid DNA by subretinal injection and electroporation of P0 mice as described previously (18, 30, 32). Briefly, the future edge of the eyelid was opened, and a pilot hole was made with a 30G needle. With a 33G blunt needle, approximately 450 nl of solution containing 1× PBS, 2 mg/ml pGrm6P-mGluR6-EGFP plasmid, and 0.1% Fast Green dye was introduced into the subretinal space using a UMP3 microinjection syringe pump and MICRO2T controller (World Precision Instruments). A plasmid expressing DsRed was included in most cases to help identify transduced cells. Electroporation was performed by placing tweezer electrodes over the eyes and delivering five 50-ms 80 V pulses, at 1 s intervals, with an ECM830 square wave electroporator (BTX Harvard Apparatus). Animals were processed for retina samples 4 to 6 weeks later.

### Immunofluorescence microscopy of retina samples

Intact eyes were fixed in 2% paraformaldehyde in PBS for ∼20 min, washed extensively in PBS, and cryoprotected in 30% sucrose in PBS overnight. Corneas were removed and eyecups with lenses were embedded in O.C.T. Compound (Tissue-Tek). Sixteen micrometer cryosections were rinsed with PBS, blocked with PBS containing 10% normal donkey serum, 5% bovine serum albumin, and 0.2% Triton X-100, and incubated overnight with 545H5 diluted to 8 μg/ml in blocking solution, followed by washing and labeling for two h with anti-mouse IgG conjugated to Alexa 647 (Invitrogen) diluted to 8 μg/ml in blocking solution. Slides were washed and mounted with Prolong Diamond (Invitrogen).

Confocal z-stacks were acquired with a Zeiss LSM880 microscope and 63× oil immersion objective. All images were acquired with identical settings. For display only, input levels were adjusted separately for each mutant to most clearly show EGFP and TRPM1 localization. OPL puncta and total intensity in maximum z-projections (ten images, 1024 × 512 pixels, z-interval 0.5 μm, x-y resolution 65.9 nm/pixel) were quantified from raw images as described (18) with modifications described below. A mask containing the OPL and part of the outer nuclear layer was created by manually removing the inner nuclear layer. Using Mathematica v.13 (Wolfram), both TRPM1 and EGFP channels in both full-field and mask images were scaled to the minimum and maximum of the full-field image, where the minimum and maximum were the average of the 100 lowest and 100 highest pixel values, respectively. A background correction was then applied to all images by subtracting the mean of the pixel values from the first row (in the outer nuclear layer). ROIs corresponding to OPL puncta were acquired from the scaled mask images using MorphologicalComponents with method Convex Hull and threshold values of 0.3 for both channels. Components containing ≥150 pixels were removed. The resulting ROIs were applied to unscaled images to obtain the intensity within OPL puncta, which was then divided by the total intensity of the unscaled full-field image.

## Data availability

The data that support the findings of this study are available by request from the corresponding author.

## Supporting information

This article contains supporting information.

## Conflict of interest

The authors declare that they have no conflicts of interest with the contents of this article.
