# Correction: Complex N-glycosylation of mGluR6 is required for trans-synaptic interaction with ELFN adhesion proteins

**DOI:** 10.1016/j.jbc.2024.107782

**Published:** 2024-09-26

**Authors:** Michael L. Miller, Mustansir Pindwarawala, Melina A. Agosto

In the Experimental Procedures section, a number of corrections should be noted:

Under the “DNA constructs” heading, the text currently reads:

“ECDs of ELFN1 (aa 1–418) and ELFN2 (aa 1–397) were fused to human Fc (from Addgene plasmid #59313, a gift from Peter Scheiffele and Tito Serafini (49)) with a GGGAAA linker in pCDNA3.1.”

It should read:

“ECDs of ELFN1 (aa 1–418) and ELFN2 (aa 1–397) were fused to human Fc (from Addgene plasmid #59313, a gift from Peter Scheiffele and Tito Serafini (49)) with a GGAAAA linker in pCDNA3.1.”

Under the “Glycosidase treatments” heading, the text currently reads:

“Glycosidase enzymes and related reagents were from Qiagen.”

It should read:

“Glycosidase enzymes and related reagents were from New England Biolabs.”

Under the “Western blotting” heading, the text currently reads:

“For SDS-PAGE, samples were mixed with 4 volumes of 5× sample buffer containing 250 mM Tris pH 6.8, 10% SDS, 50% glycerol, and 10% β-mercaptoethanol.”

It should read:

“For SDS-PAGE, samples were mixed with 1/4 volume of 5× sample buffer containing 250 mM Tris pH 6.8, 10% SDS, 50% glycerol, and 10% β-mercaptoethanol.”

Under the “Ca^2+^ mobilization experiments” section, the text currently reads:

“Cells were then incubated with 50 μl of dye loading solution (2.7 μM Fluo-4 AM (Invitrogen) in KRH with 0.06% Pluronic F-127 (Biotium)) for 1 h in the dark at room temperature.”

It should read:

“Cells were then incubated with 50 μl of dye loading solution (2.7 μM Fluo-4 AM (Invitrogen) in KRH with 0.012% Pluronic F-127 (Biotium)) for 1 h in the dark at room temperature.”

