# Supplementary material for: Complex N-glycosylation of mGluR6 is required for trans-synaptic interaction with ELFN adhesion proteins
Source: J Biol Chem. 2024 Feb 28;300(4):107119. doi: 10.1016/j.jbc.2024.107119 (PMC10973816; doi:10.1016/j.jbc.2024.107119)
Supplement: Supporting Figures S1–S5 [file mmc1.pdf]

Supporting information

Complex N-glycosylation of mGluR6 is required for trans-synaptic interaction with ELFN adhesion proteins

Michael L. Miller[1], Mustansir Pindwarawala[1], Melina A. Agosto[2,*]

[1] Medical Sciences Program, Faculty of Science, and

[2] Retina and Optic Nerve Research Laboratory, Department of Physiology and Biophysics, and Department of Ophthalmology and Visual Sciences,

Dalhousie University, Halifax, Nova Scotia, Canada

*For correspondence: melina.agosto@dal.ca

This section includes:

Supplementary Figure S1: Validation of mGluR6 mAbs in transfected HEK293T cells.

Supplementary Figure S2: WT and mutant mGluR6 partially colocalize with ER marker Sec61.

Supplementary Figure S3: Plasma membrane localization of EGFP-fused glycosylation mutants in HEK cells.

Supplementary Figure S4: Plots and linear regression lines for puncta/total measurements as a function of total in the EGFP channel.

Supplementary Figure S5: Localization of glycosylation mutants in nob3 mouse retina.

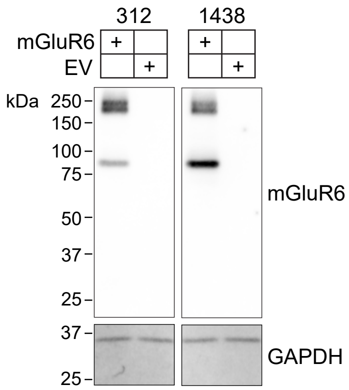

Figure S1. Validation of mGluR6 mAbs in transfected HEK293T cells. Lysates from cells transfected with WT mGluR6 or empty vector (EV) were blotted with mAb-312 or mAb-1438, followed by GAPDH antibody detected in a different channel.

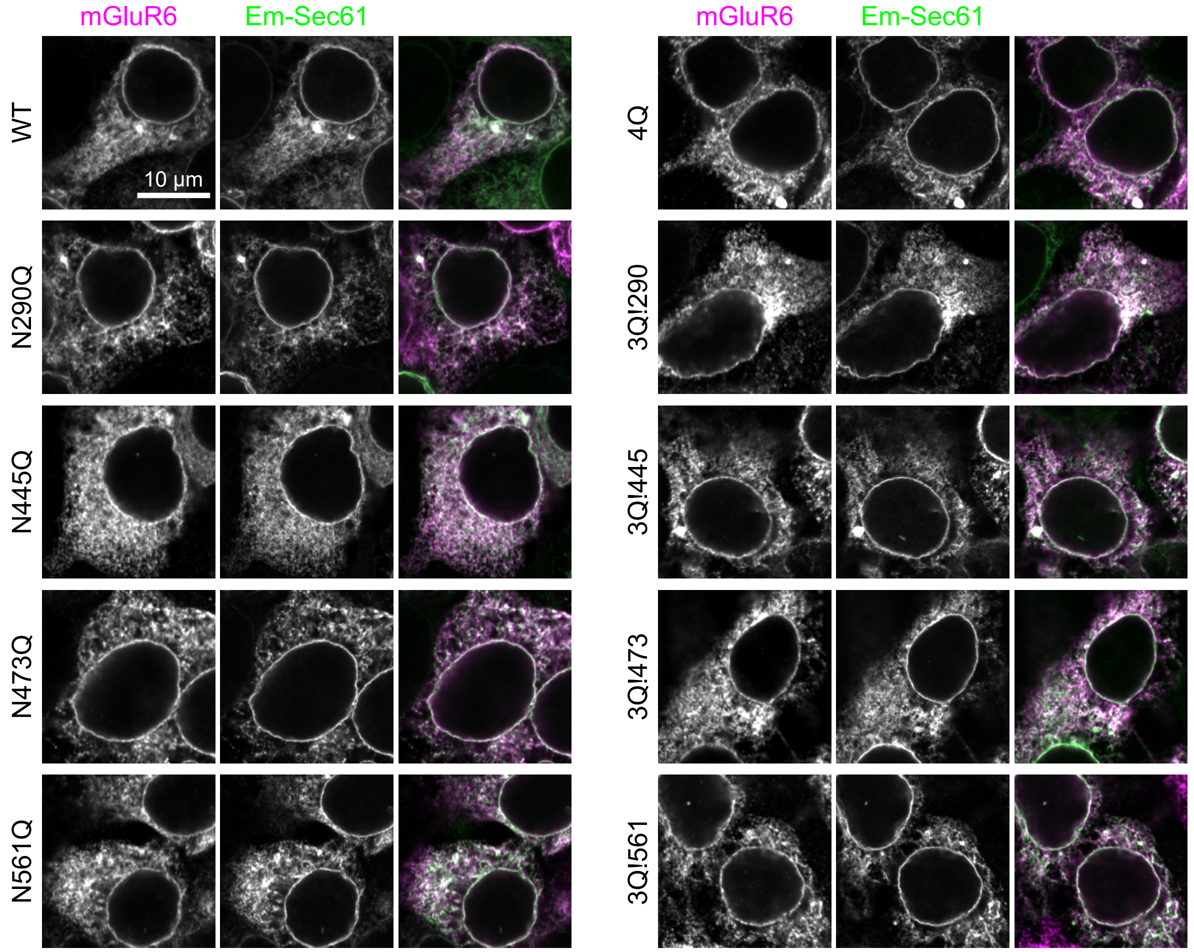

Figure S2. WT and mutant mGluR6 partially colocalize with ER marker Sec61. HEK cells co-transfected with 0.6 µg untagged mGluR6 and 0.2 µg mEmerald-Sec61 (green) were labeled in permeabilizing conditions with mAb-1438 (magenta). Images were processed independently to highlight localization; intensities should not be compared.

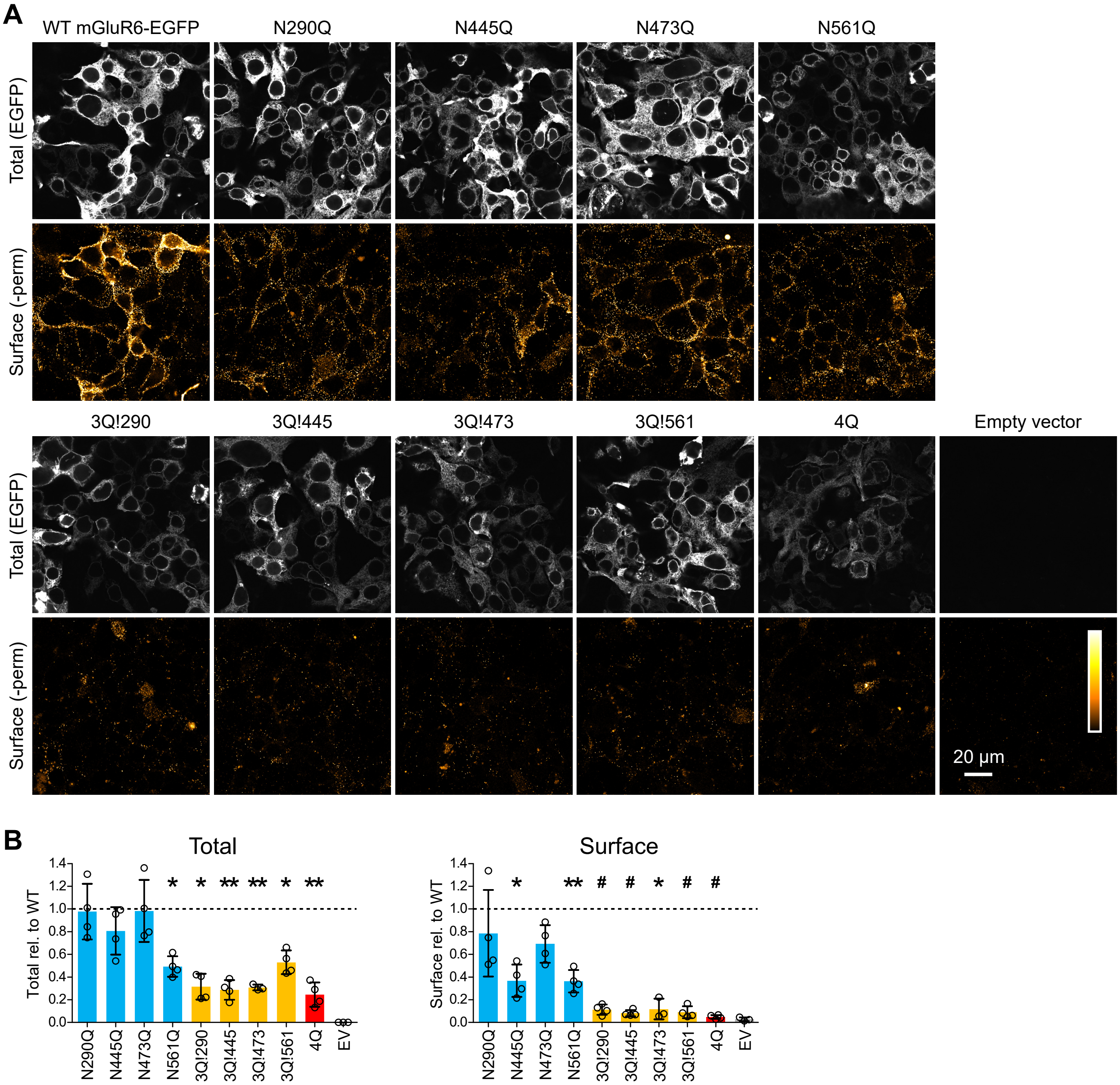

Figure S3. Plasma membrane localization of EGFP-fused glycosylation mutants in HEK cells. (A) Cells transfected with WT or mutant mGluR6-EGFP were labeled with mAb-1438 in non-permeabilizing conditions (orange). EGFP (grey) was used to measure total expression. (B) Images were analyzed as described in Fig. 5.

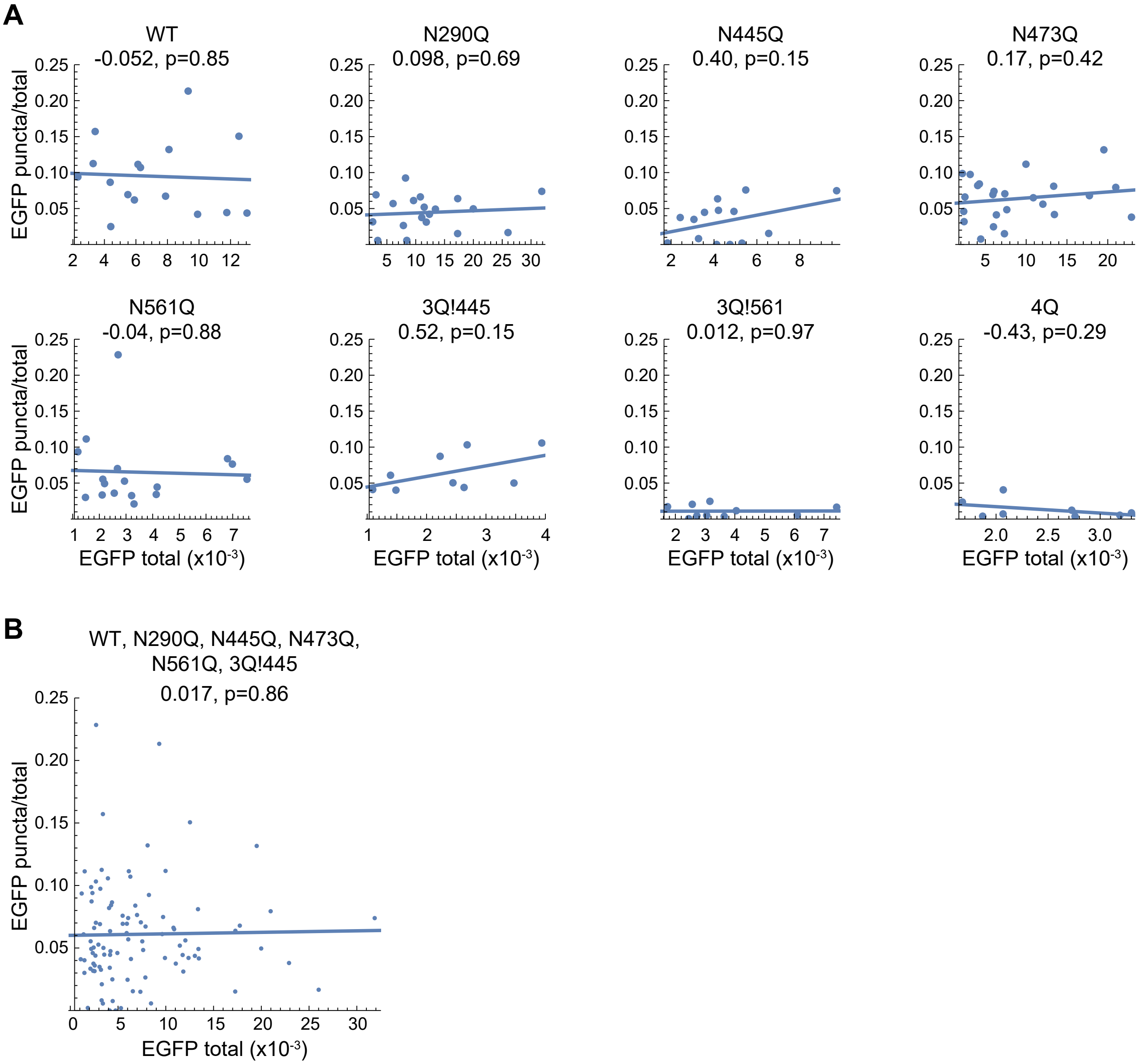

Figure S4. Plots and linear regression lines for puncta/total measurements as a function of total in the EGFP channel. Data from all images from WT CD1 mice are shown, separated by construct (A) or with puncta-forming constructs (WT, N290Q, N445Q, N473Q, N561Q, and 3Q!445) combined (B). Pearson correlation coefficients and p-values are shown above each plot.

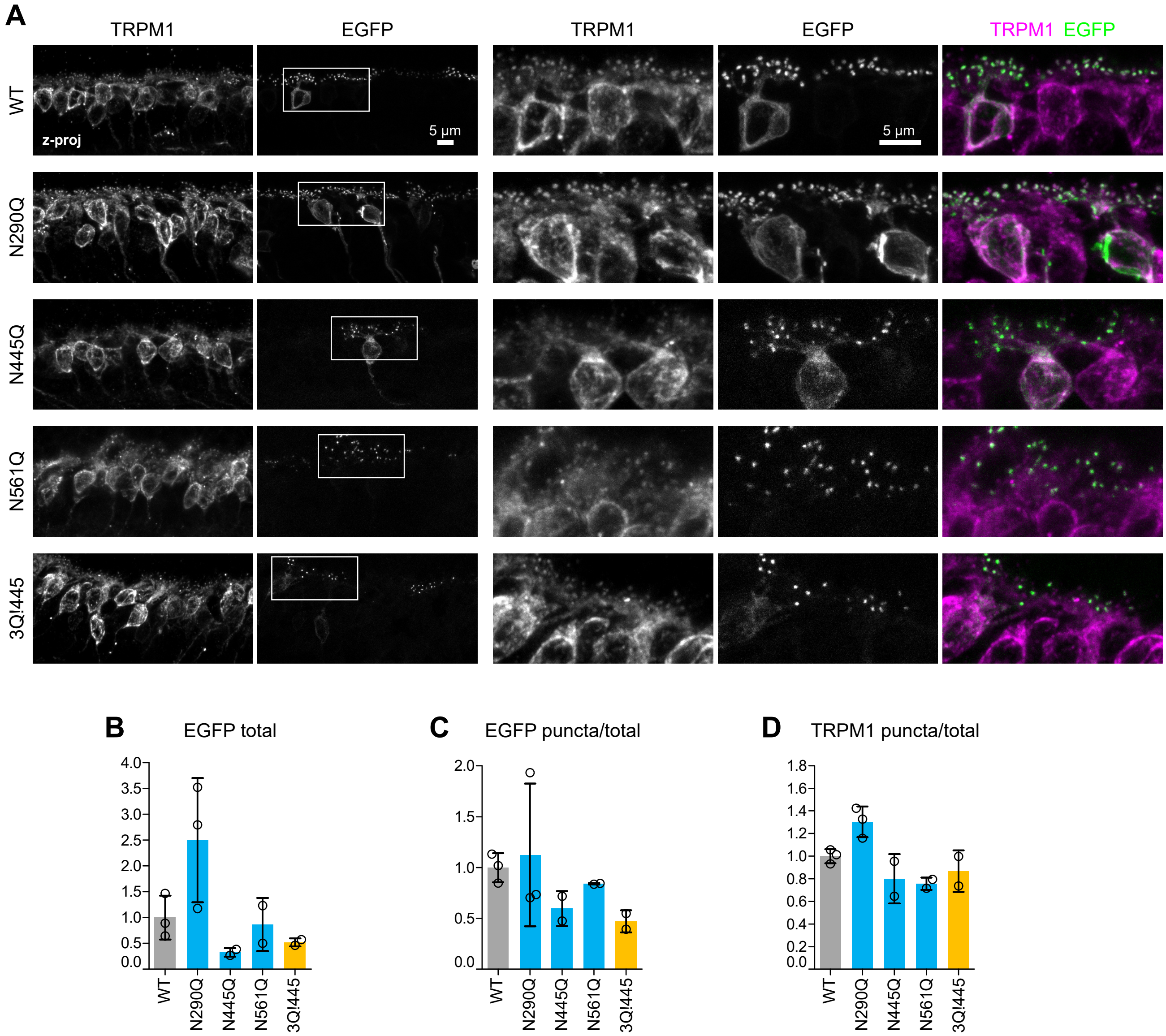

Figure S5. Localization of glycosylation mutants in nob3 mouse retina. WT or mutant mGluR6-EGFP (green) was expressed in nob3 mouse BCs by subretinal injection and electroporation. Retina sections were co-stained with TRPM1 mAb-545H5 (magenta). Images on the right are magnified views of the boxed regions. Images were processed to highlight the localization of each mutant and intensities should not be compared. (B-D) Intensity of OPL puncta in EGFP and TRPM1 channels, as well as total EGFP, were quantified as described in the Experimental procedures. Each point represents the mean of at least 3 (median n=4) images from a different animal and error bars show means ± SD of biological replicates.
